# Going beyond Environment to Context: Leveraging the Power of Context to Produce Change

**DOI:** 10.3390/ijerph17061885

**Published:** 2020-03-13

**Authors:** Robert L. Schalock, Ruth Luckasson, Karrie A. Shogren

**Affiliations:** 1 Hastings College, Hastings, NE 68901, USA; 2 Department of Special Education, University of New Mexico, Albuquerque, NM 87106, USA; ruthl@unm.edu; 3 Kansas University Center on Developmental Disabilities, University of Kansas, Lawrence, KS 69703, USA; shogren@ku.edu

**Keywords:** context, change strategies, conceptual models, human functioning, human rights, person–environment fit, quality of life, valued outcomes

## Abstract

This article discusses the processes and implications of going beyond environment to context. The article (a) provides an operational definition of context; (b) describes a multidimensional model of context that views context as being multilevel, multifactorial, and interactive; (c) describes how conceptual models of quality of life, human rights, and human functioning can be used in conjunction with the multidimensional model of context to identify opportunities and develop context-based change strategies that improve quality of life, human rights, and human functioning outcomes; and (d) describes a four-step approach to leveraging an understanding of context to produce change. The article concludes with a discussion of the advantages of and barriers to moving beyond environment to context.

## 1. Introduction and Overview

The functioning of individuals is highly influenced by their current situations. Historically, these situations were labeled as their “environments,” and an individual’s functioning was often described as the “person–environmental fit.” The concept of person–environmental fit has been very instrumental in conceptualizing disability as resulting from the interaction between a person and their environment, and implementing the supports paradigm that focuses on reducing the discrepancy between an individual’s capabilities and environmental requirements.

Despite this contribution, the concept of “person–environmental fit” is not sufficient to capture the totality of the circumstances that influence human functioning and valued outcomes. This is especially true given the significant international trends and developments impacting the disability field. These trends include (a) a commitment to the human and legal rights of persons with disabilities as reflected in the United Nations Convention on the Rights of Persons with Disabilities—UNCRPD [1,2,3]; (b) the influence of the social-ecological model of disability that emphasizes the interaction between individuals and the multiple factors at the micro-, meso-, and macrosystem that affect human functioning and personal outcomes [4]; (c) the transformative effects of the supports paradigm [5]; (d) the use of the quality of life concept as a framework for program and policy development and evaluation [6,7]; (e) the capacities approach to disability that emphasizes the core values of freedom and human dignity and the obligation of society to improve peoples’ lives [8,9,10,11]; (f) the impact of positive psychology that shifts the emphasis in disability from defectology to optimum human functioning and well-being [12]; and (g) the use of outcomes evaluation to develop outcome indicators associated with quality of life, human rights, and human functioning [13,14,15,16].

Because of these significant trends and developments, it has become necessary to move beyond the “person–environment fit paradigm” to the “context paradigm” that focuses on the interrelated conditions that surround the phenomenon being examined. Once these contextual factors and influencing conditions are understood, individuals, organizations, systems, and policy makers are in a better position to use this understanding to pursue the many opportunities that multiple stakeholders have to unfreeze the status quo and drive change through policies and practices that build contexts to enhance quality of life, human rights, and human functioning outcomes [17,18,19,20].

The purpose of this article is to discuss the processes and implications of going beyond environment to context in reference to quality of life, human rights, and human functioning. In the article, we (a) provide an operational definition of context; (b) describe a multidimensional model of context that views context as being multilevel, multifactorial, and interactive; (c) describe how conceptual models of quality of life, human rights, and human functioning can be used in conjunction with a multidimensional conceptual model of context to guide the identification of opportunities and the development of context-based change strategies that enhance personal/valued outcomes; (d) describe a four-step approach to leveraging an understanding of context to produce change; and (e) discuss the advantages of and barriers to moving beyond environment to context.

## 2. Operational Definition of Context

The concept of context allows one to capture more of the complexity in the lives and functions of individuals by incorporating the micro-, meso-, and macrosystems, as well as multiple factors and interactions that influence outcomes. We define context as “*a concept that integrates the totality of circumstances that comprise the milieu of human life and human functioning*” [21] and propose that context can be viewed as an independent variable, an intervening variable, and an integrative construct.

- As an *independent variable*, context includes personal and environmental characteristics that are not usually manipulated, such as age, language, culture and ethnicity, gender, and family.
- As an *intervening variable*, context includes organizations, systems, and societal policies and practices that can be manipulated to enhance human functioning and personal outcomes.
- As an *integrative concept*, context provides a framework for (a) describing and analyzing aspects of human functioning such as personal and environmental factors, planning systems of supports, and developing disability policy; and (b) delineating the factors that affect, both positively and negatively, human functioning.

## 3. A Multidimensional Model of Context

**Figure 1:**
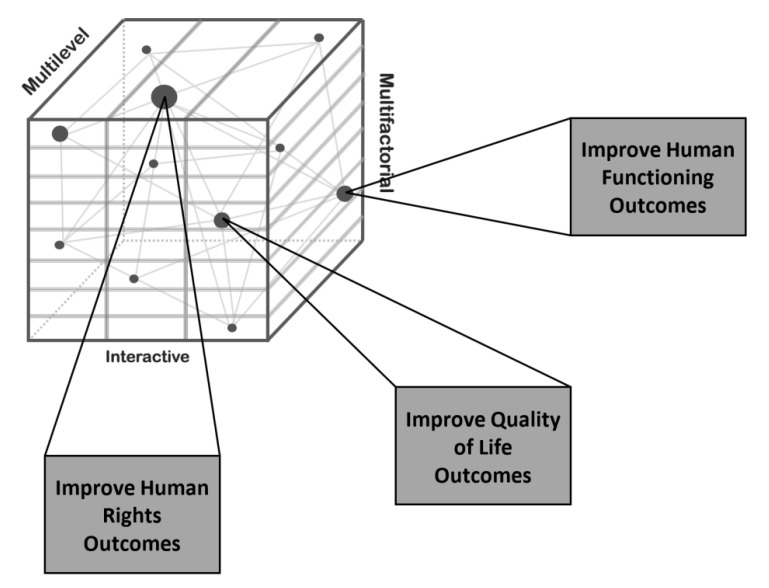
A Multidimensional Model of Context.

Based on our most recent work [22] we have developed a multidimensional model of context that is shown in Figure 1. This multidimensional model conceptualizes context as being multilevel, multifactorial, and interactive. Each of these components is described next.

### 3.1. Multilevel

This component of the multidimensional model identifies and describes the ecological systems within which people live, are educated, work, and recreate. The person and these environments interact over time and thereby influence personal outcomes differentially over time. The *micro level* typically includes immediate social settings, such as the person’s family, close friends, and advocates. The *meso level* typically includes one’s neighborhood and community, and the organizations providing services and supports. The *macro level* typically includes the larger policy context and supports delivery system, and the overarching pattern of culture, society, country, or social-political influences [23].

### 3.2. Multifactorial

This component of the model identifies and describes the potentially influential factors within the ecological systems within which people live, are educated, work, and recreate. Some of these factors (e.g., age, language, culture and ethnicity, and family structure) are not typically manipulated or changed to enhance outcomes, but need to be understood in order to identify opportunities and develop context-based change strategies. Other contextually based influencing factors can be manipulated or changed to achieve policy goals and enhance personal outcomes. Based on the authors’ work to date, Table 1 lists a number of these contextually-based factors that influence outcomes related to quality of life, human rights, and and/or human functioning.

### 3.3. Interactive

This component of the multidimensional model of context identifies and describes the variety of ways in which levels and factors interact to influence personal outcomes. *An interaction is a reciprocal action or influence that occurs between multilevel and multifactor contextual variables.* These interactions are denoted by dots in Figure 1, and vary in size depending on their relevance and importance to an individual. The dots also represent a *connection point* between the three elements of the multidimensional model of context (multilevel, multifactor, and interaction) with parallel elements of quality of life, human rights, and human functioning conceptual models (to be described in the following section). *These connection points allow service/supports providers and consumers to successfully leverage an understanding of context to identify opportunities and develop change strategies to improve quality of life, human rights, and/or human functioning outcomes* [24].

In the following section, we describe the elements of conceptual models related to quality of life, human rights, and human functioning, and align these elements to the elements of the multidimensional model of context presented in Figure 1. Based on this alignment and an understanding of context, policy makers, service/support providers, and consumers can identify opportunities and develop context-based change strategies to improve quality of life, human rights, and human functioning outcomes. In Section 6 we explain a four-step approach that facilitates this process.

## 4. Conceptual Models That Facilitate the Identification of Opportunities and the Development of Context-Based Change Strategies

Identifying opportunities and selecting context-based change strategies to improve quality of life, human rights, and human functioning outcomes is facilitated by using a conceptual model of each of these three targeted areas. A conceptual model is a representation of a phenomenon, such as quality of life, human rights, or human functioning, and incorporates context-based concepts that are used to help people understand the phenomenon and use that understanding to bring about change. As used in this article, the major purposes of a conceptual model are to (a) organize and synthesize information about quality of life, human rights, and human functioning that will facilitate identifying opportunities to improve personal outcomes; and (b) guide the selection of context-based change strategies to enhance this goal. The use of a clearly articulated conceptual model also facilitates integrating theoretical perspectives on intellectual disability that include biomedical, psychoeducational, sociocultural, and justice [25], selecting relevant evidence-based strategies [26,27] and defining operational disability-related constructs and associated outcome measures [22].

A conceptual model can be research based (as in the case of the quality of life and human functioning models) or a consensus document (such as the *United Nations Convention on the Rights of Persons with Disabilities*; UNCRPD). Examples of each, including their key features, are presented next.

### 4.1. Quality of Life Conceptual Model

The concept of quality of life (QOL) has emerged internationally as a value-based framework for opportunity development, supports provision, and outcomes evaluation [28]. Although QOL is defined and conceptualized in multiple ways, two conceptual models are frequently referenced. The first is that proposed by the World Health Organization that conceptualizes quality of life as a multidimensional phenomenon encompassing physical, mental, and social functioning and well-being [7]. The six core QOL dimensions operationalizing this conceptual model are physical health, psychological state, level of independence, social relationships, environment (e.g., financial resources and opportunities), and spirituality or religion or personal beliefs. The second commonly referenced QOL conceptual model is that proposed by Schalock, Verdugo, Gomez, and Renders [29]. These authors/investigators conceptualize QOL as a multidimensional phenomenon that is composed of domains that reflect one’s personal well-being. These domains include personal development, self-determination, interpersonal relations, social inclusion, rights, emotional well-being, physical well-being, and material well-being. These domains are influenced by systems of supports (i.e., choice and personal autonomy, inclusive environments, generic supports, and specialized supports) that can act as moderators or mediators in influencing QOL outcomes [30].

Quality of life dimensions/domains and supports interact due to the reciprocal action or influence that occurs between multilevel and multifactorial contextual variables. Specifically:

- *Multilevel factors* influence QOL outcomes at the micro-level through factors such as personal and family attitudes and expectations about the person’s disability, strengths, and limitations, and the availability of resources, services, and supports. At the meso level, QOL outcomes are affected by legislative and statutory opportunities and systems of supports provided at the community level. At the macro level, QOL outcomes are influenced by societal and cultural concepts of disability, with associated stereotypes and attitudes, and the resources a society devotes to quality of life enhancement.
- *Multifactors* influence QOL outcomes through the influencing factors listed in Table 1 and the provision of systems of supports. As summarized in Table 1, these contextually based influencing factors can occur at the micro-, meso-, and/or macrosystem level.
- *Interactions* occur between multilevels and multifactors and affect QOL outcomes through (a) *quality of life* facilitating conditions, such as participation in the community, promoting a sense of belonging, maximizing capabilities, freedom to engage in major life activities, commitment to goals that are important to the person or family, and respect for and enhancement of differences; and (b) *support facilitating conditions*, such as the availability and accessibility of supports, safe and secure environments, information about systems of support, competent support providers, consistency of supports provision, coordination and management of supports, and collaboration among professionals and support providers.

The alignment of the quality of life conceptual model with the multilevel, multifactor, and interactive elements of the multidimensional model of context depicted in Figure 1 can be used to (a) identify opportunities related to enhancing quality of life by understanding the multiple systems that affects one’s quality of life, the multiple factors that impact one’s QOL, and how multiple-levels and multiple factors interact in terms of quality of life and support facilitating conditions; and (b) to develop context-based change strategies to enhance one’s quality of life. Specific details about this process are discussed in Section 6.

### 4.2. Human Rights Model

A human rights conceptual model based on the *United Nations Convention of the Rights of Persons with Disabilities* provides the framework to identify opportunities and develop context-based strategies to maximize peoples’ human rights [2,3,31,32]. The convention calls for a fundamental reappraisal of policy and practice by society, governments, members of professional and voluntary organizations, service and support providers, and individuals regarding peoples’ human and legal rights. The parameters of the UNCRPD can be aligned with the multiple elements of the multidimensional model of context presented in Figure 1. Specifically:

- *Multilevels* are reflected in the obligations that signatories are committed to. These obligations involve modification or repeal of laws, customs, and practices that discriminate directly or indirectly against people with disabilities; inclusion of disability in all relevant policies; reframing any practice inconsistent with the UNCRPD; consulting with people with disabilities and their organizations in implementing the UNCRPD; and making “reasonable accommodation” to all relevant aspects of the environment so as to enable people with disabilities to exercise their rights.
- *Multifactors* are reflected in the convention’s General Principles that encompass respect for inherent dignity and individual autonomy; equality and nondiscrimination; full and active participation and inclusion in society; respect for differences and acceptance of persons with disabilities as part of humanity; accountability; equality between men and women; and respect for the evolving capabilities of children with disabilities and the right to preserve their identities.
- *Interactions* that occur between these multilevels and multifactors have been selected and mandated by the international community to promote the articles identified in the UNCRPD. These convention articles/outcomes relate to equality and non-discrimination (Article 5), accessibility (Article 9), right to life (Article 10), equal recognition before the law (Article 12), liberty and security of person (Article 14), freedom from exploitation, violence, and abuse (Article 16), liberty of movement and nationality (Article 18), living independently and being included in the community (Article 19), personal mobility (Article 20), freedom of expression and opinion and access to information (Article 21), respect for privacy (Article 22), education (Article 24), health (Article 25), habilitation and rehabilitation (Article 26), work and employment (Article 27), adequate standards of living and social protection (Article 28), and participation in political and public life (Article 29).

Parallel with the quality of life conceptual model, the human rights conceptual model described above aligns elements of the UNCRPD with the element of the multidimensional model of context depicted in Figure 1. This alignment can be used to identify opportunities and develop context-based change strategies to maximize peoples’ human rights. As described in Section 6, this involves targeting the signatories’ obligations, the UNCRPD general principles, and the convention’s articles.

### 4.3. Human Functioning Conceptual Model

The human functioning conceptual model described in this article has it origin in the *International Classification of Functioning, Disability, and Health* [33]. That model, which is based on a definition of health as a state of complete physical, mental, and social well-being, conceived human functioning as an interactive person–environmental process, and disability as problems in functioning. Furthermore, the ICF model viewed human functioning as resulting from the complex interactions among health conditions (disease and disorder), body structures and functions (impairments), activities (activity limitations), participation (participation restrictions), environmental factors (barriers, hindrances), and personal factors. In the model, personal and environmental factors were referred to as “contextual factors” [34].

The human functioning model presented below is a logical extension of the ICF model and incorporates recent work in (a) operationalizing health in terms of human functioning, and (b) applying a human functioning approach to disability. In reference to operationalizing health in terms of human functioning, Stucki and Bickenbach advocate using the concept of human functioning as a measureable indicator of health [16]. Such use, according to the authors, clarifies, conceptually and quantitatively, the link between health and well-being. Building on the ICF model, the authors explain (p. 1790) how functioning is understood biochemically and in terms of the functions and structures of the body and the intrinsic health capacity of a person to perform simple and complex activities, as well as the actual performance of those activities in interaction with features of the person’s physical and social environments [16].

In reference to applying a human functioning approach to disability, Luckasson and Schalock defined such an approach as involving “a systems perspective towards understanding human functioning that includes human functioning dimensions, interactive systems of supports, and human functioning outcomes” [35]. From a systems or logic model perspective, the human functioning conceptual model is operationalized through its *input component*, which includes the human functioning dimensions of intellectual functioning, adaptive behavior, health, participation, and context; its *throughput component* that incorporates systems of supports; and its *output component* that encompasses human functioning outcomes related to socio-economic status, health status, and subjective well-being. These outcome categories encompass both the six dimensions of the WHO-QOL Scales (WHO, 1997), and the eight domains of the Schalock et al. QOL model discussed earlier [29]. The human functioning conceptual model presented in this article (a) is consistent with the model incorporated into the 10th and 11th editions of the American Association on Intellectual and Developmental Disabilities Terminology and Classification Manuals [36,37]; and (b) reflects our increased understanding of the multidimensionality of human functioning and the significant progress in our understanding of support strategies and outcomes evaluation [4,28].

As with the QOL and human rights conceptual models described previously, components of the human functioning model described above can be aligned with the three elements of the multidimensional model of context depicted in Figure 1. Specifically:

- *Multilevels* encompass the individual, community and service/support organizations, and governments and society.
- *Multifactors* relate to the individual’s status vis-à-vis intellectual functioning, adaptive behavior, health, participation, and context, and the level and type of supports available to the individual [32,34].
- *Interactions* occur between the multiple levels and multiple factors listed above. Human functioning is improved when interactions are targeted and supported across levels and factors.

## 5. Using the Multidimensional Model of Context to Identify Opportunities and Developi Context-Based Change Strategies

In this section of the article we describe in more detail how an understanding of elements of a multidimensional model of context and analogous elements of a conceptual model of quality of life, human rights, or human functioning can be used to identify opportunities and develop context-based change strategies. As depicted in Figure 1 and described in Table 1, opportunities result from the interaction of the *ecological systems* within which people live, learn, work, and recreate, and the *context-based influencing factors* within these ecological systems. The better one understands these systems, factors and conceptual models, the more able one is to identify opportunities and develop context-based strategies to improve peoples’ quality of life, human rights, and human functioning outcomes.

### 5.1. Identifying Opportunities

The interactive property of context was depicted in Figure 1 as “dots” of various sizes, which reflect the potential relevance to a proposed change, and the importance of the specific interaction to an individual. Each dot also represents a *connection point* between the elements of the multidimensional model of context and respective elements of a quality of life, human rights, or human functioning conceptual model. These interactions create the opportunities for intervention and change. Examples include increasing one’s social inclusion in school, increasing opportunities to live independently in the community, or improving one’s health status.

Once these opportunities for change are identified, they need to be evaluated as to whether (a) they reflect the person’s values, personal goals, and personal desires; (b) are examined as to their cultural relevance and technical feasibility; and (c) consistent with policy goals that have a high priority for a society or organization. As described next, once these opportunities are identified, specific context-based support strategies can be developed to produce change.

### 5.2. Developing Context-Based Change Strategies

A specific interaction (i.e., “opportunity”) can be systematically influenced and changed by manipulating one or more contextually-based influencing factors. Context-based change strategies may involve multiple change mechanisms, such as adding systems of supports elements, advocating for policy changes, or changing personal circumstances and skills. Examples include (a) legislative changes over the last two decades that have strengthened the relation between supported employment initiatives (factor at the macro level) and organizations implementing supported employment opportunities (factor at the mesosystem); (b) the introduction of decision making supports legislation (factor at the macro-level) with the implementation of a new research-based program for supported decision making (meso-level), which has increased decisions being made by the individual with a disability rather than decisions being made for the person (factor at the micro level); and (c) living in a country where the UNCRPD has been adopted and where there is a commitment to the inherent dignity and human rights of people with disabilities through rights-centered policies and practices, community-based services and supports, high quality health services, and the provision of individualized systems of supports. The context-based change strategy or strategies selected should be based on:

- A contextual analysis that identifies factors that hinder change and forces that facilitate change, and a context-based change model that approaches changes from the perspective of a quality improvement loop that involves analysis, planning, doing, and evaluation [24,38,39].
- An understanding of the contextually-based factors that influence personal outcomes (see Table 1 for examples).
- The multilevel/multifactor parameters of the previously discussed multidimensional model of context and the quality of life, human rights, and human functioning conceptual models.

The following section describes a four-step systematic approach that can be used to develop context-based change strategies that leverage an understanding of context to produce change. The approach involves identifying a needed change, identifying interactions, identifying the levels and factors influencing the opportunity for change, and developing a context-based strategy. 

## 6. A Four-Step Approach to Leveraging an Understanding of Context to Produce Change

### 6.1. Step 1: Identify a Desired and Needed Change

This first step involves identifying, in collaboration with the person, a needed change (which represents “an opportunity”) in the person’s life, an organization or community’s ability to support people, or a government’s desire to provide supports. Using a conceptual model such as that described previously on context, quality of life, human rights, or human functioning facilitates this first step. Specifically, and as described previously in reference to each conceptual model, the alignment of elements of the respective conceptual model with the multilevel, multifactor, and interactive elements of the multidimensional model of context (see Figure 1) allows one to identify interactions (see Step 2) and potential contextual factors influencing the opportunity for change (see Step 3)

### 6.2. Step 2: Identify Interactions That Potentially Influence Desired or Needed Change

This step focuses on “opportunity development” and requires the analysis of the reciprocal action or influence that occur between multilevel and multifactor contextual variables. Conceptual models, such as those for quality of life, human rights, and human functioning, are also useful in this step both to help individuals, service/ support providers, and policy makers develop context-based change strategies and to select outcome indicators to evaluate the current attainment level of the identified needed/desired change [14,40].

Interactions are denoted by dots in Figure 1, and vary in size depending on their relevance to the proposed change and importance to the individual, organization, community, or society. The dots also represent a connection point between the three elements of the multidimensional model of context with parallel elements of the quality of life, human rights, and human functioning conceptual models described previously. As part of the identification process, potential targeted interactions should be examined as to their cultural relevance and technical feasibility and their consistency with policy goals that have a high priority for an organization or society. The interactions identified for needed change should also be based on “the voice of the person” and reflect the individual’s values, personal goals, and personal desires.

### 6.3. Step 3: Identify the Levels and Factors Influencing the Opportunity for Change

Levels and factors influencing an opportunity/desired change can be identified either by using Table 1 that summarizes literature-based systems-level contextual factors that influence personal outcomes, or by analyzing the multilevel and multifactor elements described previously that are associated with a quality of life, human rights, or human functioning conceptual model. The identified levels and factors are considered as intervening variables that can be manipulated to produce change [21], and act as moderator or mediator variables in the production of change [29]. Examples include (a) micro-system level factors, such as personal strengths/assets, choices/opportunities, and the availability of systems of supports; (b) meso-system level factors, such as organization policies and practices; and (c) macro-system level factors, such as community access and participation, community-based alternatives, living and employment supports, justice and fairness in the legal system, and societal attitudes, public policies, and system practices.

### 6.4. Step 4: Develop a Context-Based Change Strategy

Selecting a context-based change strategy follows the identification of the levels and factors influencing the opportunity for desired change. Context-based change strategies are used to bring about change in the desired interaction/needed change area. Potential change strategies are also selected based on their potential to promote adoption and increase stakeholder participation. These strategies typically involve disability policy changes, organization transformation strategies, and/or providing systems of supports.

- *Disability policy changes* involve *an integrated approach* to disability policy development, implementation, and evaluation [41], and *a cross-cultural approach* that uses a contextual analysis, emphasizes a value-based approach, aligns the service delivery system both horizontally and vertically, and engages in a partnership in policy implementation [42].
- *Organization transformation* involves *transformation pillars* that include values, self-evaluation, critical thinking skills, and innovation, and *transformation strategies* that include analyzing environments, aligning organization practices, incorporating a balanced approach to performance management, integrating ecological systems, and employing strategic execution [39].
- *Systems of supports* emphasize the provision of supports that involve choice and personal autonomy, inclusive environments, generic supports, and specialized supports [4].

Table 2 shows how the four steps just described can be aligned to bring about desired change in quality of life, human rights, and human functioning outcomes.

## 7. The Advantages of and Barriers to Going beyond Environment to Context

### 7.1. Advantages

Thus far in this article, we have (a) described how a multidimensional model of context can be used to identify opportunities for needed change and implement context-based change strategies; (b) applied this heuristic to the areas of quality of life, human rights, and human functioning; and (c) outlined a four-step approach to leveraging an understanding of context to produce desired change. Although each of these activities is potentially complex since they involve new concepts and terminology that require both changing one’s thinking and understanding the multidimensional properties of context, going beyond environment to context has definite advantages to the field. Chief among these are that the approach to context described in this article provides:

- A broader and more complete picture of the complexities of the lives of people with disability.
- A challenge to the field to systematically explore contextually-based levels and factors that influence human functioning and valued personal outcomes.
- An understanding of the power and importance of the interaction (i.e., reciprocal action or influence) that occurs between multilevel and multifactor contextual variables.
- An analytic framework for identifying and prioritizing opportunities for desired or needed change, and context-based change strategies that honor and reflect the complexities of people’s lives.
- A systematic approach that is transparent to all parties and facilitates replication and application.

### 7.2. Barriers

Barriers will be encountered by those advocates, policy makers, practitioners, or researchers who either want to apply the approach to context described in this article, or to extend the systematic approach described to areas other than quality of life, human rights, and human functioning. These barriers potentially relate to (a) the requirement that people who “are committed to” the person–environment model will need to broaden their perspective; (b) the complexity of the contextual analyses used to identify and understand reciprocal interactions; (c) the required participation of more individuals who can contribute to the more complex analyses; (d) the need to understand international trends and developments, such as the UNCRPD, social-ecological model of disability, support paradigm and systems of supports, QOL concept and its application, capacities approach, and outcomes evaluation; (e) the potential exposure of decision making processes that were previously more private; and (f) the need to prepare and support people so that they can effectively participate in the four-step approach to leveraging an understanding of context to produce change.

As stressed by one of the Reviewers, a significant challenge of going beyond environment to context is to overcome the “operationalization hurdle” faced by previous approaches, such as the person–environmental fit concept, the capacities approach, or the social-ecological model of disability. The advantage and contribution of the multidimensional model of context presented in Figure 1 and discussed in this article is that an understanding of the multilevel, multifactor, and interactive properties of context provides an *operationalization framework* to describe and analyze the impact of personal and environmental factors on valued outcomes, including those related to quality of life, human rights, and human functioning. As discussed more fully in Gomez et al., Schalock, Gomez et al., and Schalock, Verdugo et al., five steps are involved in developing and implementing an operationalization framework [26,27,29]. First, define the practices in question. Exemplary practices were identified in Table 1 in reference to contextually based factors that influence quality of life, human rights, and human functioning outcomes. Second, select outcome areas and outcome indicators. This step involves incorporating commonly used outcome areas, such as quality of life domains, human rights areas, and human functioning dimensions, and relating the specific outcome areas to measurable outcome indicators that can be assessed in reliable and valid ways. Third, gather evidence. Evidence gathering involves (a) employing an evidence-gathering strategy that is consistent with the question(s) asked, the practices being evaluated, statutory/regulatory parameters, the constituents involved, and the available expertise; (b) defining operationally the strategies; and (c) demonstrating implementation fidelity. Fourth, establish the credibility of the evidence, which involves evaluating the obtained evidence in terms of its quality, robustness, and relevance. Fifth, evaluate the relation between practice(s) and outcome(s). This final step requires determining if there is substantial evidence that the outcome was caused by the practice; it has been demonstrated that the intervention clearly leads to the outcome, and/or the intervention has a plausible rationale to explain why it should work and with whom.

Although these barriers may be seen as daunting and pose significant challenges, they also represent opportunities to use an understanding of context to unfreeze the status quo and drive change to enhance valued quality of life, human rights, and human functioning outcomes for people with disabilities. Overcoming these barriers is also a primary responsibility of disability organizations and systems as they continue their efforts to provide services and supports that enhance valued outcomes for people with disabilities and their families. To these ends, we offer the following guidelines. First, clearly identify the targeted area (i.e., “the phenomenon under consideration”). Second, integrate the elements or components of a valid conceptual model of the phenomenon under consideration with the multilevel, multifactor, and interactive elements of the multidimensional model of context presented in Figure 1. This will allow one to identify multilevel, multifactor, and interactive elements that can be used to select opportunities for desired change, and the factors influencing the effectiveness of the context-based change strategies selected and implemented. Third, use a systematic approach, such as the previously described four-step process, to leverage an understanding of context to produce change. Fourth, evaluate the change produced. The evaluation should focus on the status of the identified needed change (see Table 2 for examples), and incorporate the five-steps involved in the operationalization framework described above.

## 8. Conclusions

In conclusion, the field of disability in general, and the field of intellectual and developmental disability specifically, has gone beyond the person–environment paradigm to a multidimensional contextual paradigm with its related terminology and application. This shift is in line with current international trends and developments and the ongoing realities of the lives of people with a disability that involve multiple levels, factors, and interactions that either facilitate or hinder change in their lives. The multidimensional aspects of context need to be understood in order to unfreeze the status quo and drive valued change through policies and practices that build contexts to improve quality of life, human rights, and human functioning outcomes. As reflected in this article, “it is not just about environment anymore”.

## Figures and Tables

**Table 1:** Factors that influence quality of life, human rights, and/or human functioning outcomes.

Microsystem Level	Mesosystem Level	Macrosystem Level
-Personal strengths/assets -Health condition -Limitations in intellectual functioning and/or adaptive behavior -Social networks -Family involvement -Choices/opportunities -Decision making supports -Self-advocacy -Augmentative communication systems -Information and assistive technology devices -Natural supports	-Alignment of services and supports to personal goals and assessed support needs -Person-centered planning -User-friendly personal support plans -Environmental accommodation -Organization policies that emphasize improved quality of life, human rights, and/or human functioning.	-Opportunities for increased interdependence, productivity, and community integration -Community access and participation -Community-based alternatives -Living and employment supports -Justice and fairness in the legal system -Legal rights and protections -Transportation availability -Societal attitudes, public policies, and system practices

**Table 2:** The four-step approach to bring about change.

Identified Needed Change (Step 1)	Identified Multilevel and Multifactor Interactions and Influencing Factors (Steps 2 and 3)	Selected Context-Based Change Strategies (Step 4)
Increase social inclusion in school (quality of life)	-Micro level: attitudes of peers without disabilities (interpersonal level) -Meso level: degree to which the school’s leadership prioritizes social inclusion (organization level)	-Implement a program that promotes social inclusion of students with disabilities at multiple levels using a school-wide approach
Increased opportunity to live independently and being included in the community (UNCRPD Article 19)	-Macro level: restrictive laws and customs regarding persons with disabilities -Meso level: lack of community-based residential options and living supports	-Modify housing codes -Work with professionals and self-advocates to demonstrate strengths and capacities -Implement community living options -Provide systems of supports (e.g., “supported living”)
Improve health status (human functioning)	-Meso level: poor access to health care system -Micro level: lifestyle and health related beliefs that are incompatible with proper nutrition	-Interface person with the health care system (e.g., community clinic) -Provide information and monitoring about proper nutrition -Establish a productive interaction between the person and the community clinic to monitor nutrition and lifestyle changes
